# Diagnostic Accuracy of Bisphosphonate Scintigraphy in Glu54GlnATTR Cardiomyopathy

**DOI:** 10.3390/jcm14113734

**Published:** 2025-05-26

**Authors:** Claudiu Stan, Gabriela Neculae, Robert-Daniel Adam, Andreea Jercan, Sorina-Nicoleta Badelita, Mirela-Ramona Draghici, Camelia Dobrea, Sebastian Onciul, Razvan Capşa, Cristina Chirion, Dan Stanescu, Cipriana Stefanescu, Irena-Cristina Grierosu, Teodor-Marian Ionescu, Ana-Maria Statescu, Mihai Gutu, Alessia Argiro, Francesco Cappelli, Daniel Coriu, Ruxandra Jurcuţ

**Affiliations:** 1 Faculty of Medicine, “Carol Davila” University of Medicine and Pharmacy, 020021 Bucharest, Romania; gabriela.neculae@drd.umfcd.ro (G.N.); ruxandra.jurcut@umfcd.ro (R.J.); 2 Department of Nuclear Medicine and Ultrasonography, Fundeni Clinical Institute, No. 258, 022328 Bucharest, Romania; 3 Department of Cardiology, Expert Center for Rare Genetic Cardiovascular Diseases, “Prof. Dr. C.C. Iliescu” Emergency Institute for Cardiovascular Diseases, 022328 Bucharest, Romania; 4 Department of Hematology, Fundeni Clinical Institute, No. 258, 022328 Bucharest, Romania; 5 Department of Neurology, Fundeni Clinical Institute, No. 258, 022328 Bucharest, Romania; 6 Department of Pathological Anatomy, Fundeni Clinical Institute, No. 258, 022328 Bucharest, Romania; 7 Department of Cardiology, Floreasca Emergency Clinical Hospital, 014461 Bucharest, Romania; 8 Department of Radiology, Fundeni Clinical Institute, No. 258, 022328 Bucharest, Romania; 9 Faculty of Medicine, “Grigore T. Popa” University of Medicine and Pharmacy, 700115 Iasi, Romania; 10 Department of Nuclear Medicine, “Prof. Dr. Valeriu Rusu” Emergency Clinical Hospital “St. Spiridon”, 700111 Iasi, Romania; 11 Department of Nuclear Medicine, Suceava Emergency Hospital “Sf. Ioan Cel Nou”, 720224 Suceava, Romania; 12 Faculty of Medicine and Biological Sciences, “Stefan Cel Mare” University of Medicine, 720229 Suceava, Romania; 13 Department of Experimental and Clinical Medicine, University of Florence, 50134 Florence, Italy; 14 Tuscan Regional Amyloidosis Referral Center for Cardiac Amyloidosis, Careggi University Hospital, 50134 Florence, Italy

**Keywords:** cardiac amyloidosis, transthyretin, bisphosphonate scintigraphy, bone scan, ATTR, Glu54Gln, H/CL ratio, H/L ratio

## Abstract

**Background:** Bisphosphonate scintigraphy (BS) is a recognized tool for diagnosing amyloid transthyretin cardiomyopathy (ATTR-CA). However, its sensitivity for rare transthyretin (TTR) variants, like Glu54Gln, remains underexplored. **Methods**: This was a retrospective descriptive study including all known patients with the Glu54Gln variant diagnosed between 2017 and 2023 in Romania, aiming to evaluate the diagnostic performance of BS in Glu54Gln ATTR–CA. **Results**: All symptomatic patients (*n* = 22) with histologically confirmed ATTR-CA had positive BS results (100% sensitivity). No false negatives were observed in asymptomatic carriers (*n* = 4). The Perugini visual score correlated with disease severity, with grade 3 scores associated with advanced cardiac involvement. We proposed a new parameter, heart-to-liver-uptake (H/L) ratio, which proved a strong positive correlation with both the heart-to-contralateral-uptake (H/CL) ratio (R^2^ = 0.768, *p* < 0.001) and interventricular septum thickness (R^2^ = 0.584, *p* < 0.001) and a weak correlation with the global longitudinal strain (R^2^ = 0.212, *p* = 0.023). **Conclusions**: BS demonstrates high diagnostic accuracy for Glu54GlnATTR-CA, underscoring its utility in early diagnosis and clinical management. The H/L ratio presents a novel approach to semiquantitative analysis of bisphosphonate uptake in cardiac amyloidosis, potentially addressing key limitations of the traditional H/CL ratio.

## 1. Introduction

Amyloidosis is the largest group of protein misfolding diseases characterized by the extracellular deposition of insoluble fibrils, leading to organ dysfunction. In systemic amyloidosis, circulating proteins may deposit in multiple organs, such as the heart, kidneys, spleen, liver, or nervous system. Based on the type of the precursor protein, there are different types of amyloidosis described in the literature with either systemic or localized deposition [1].

The two types of transthyretin cardiac amyloidosis (ATTR-CA) are the hereditary or variant form (ATTRv-CA) and the wild type (ATTRwt-CA). With 146 known variants of the TTR gene, hereditary transthyretin cardiomyopathy is a rare autosomal dominant condition with varied course and clinical manifestations [2]. The Glu54Gln mutation, prevalent in northeastern Romania, is associated with a mixed phenotype of cardiac and neurological manifestations [3].

Both echocardiography and cardiac MRI are key imaging techniques used to raise the suspicion of cardiac amyloidosis. However, bisphosphonate scintigraphy is the only non-invasive imaging method that can definitively confirm a diagnosis of transthyretin (ATTR) amyloidosis, after ruling out light chain (AL) amyloidosis. This diagnostic approach has been established in previous studies and is endorsed by both the European and American cardiology societies [4,5,6].

Scintigraphy using technetium-labeled (99mTc) bisphosphonate tracers has emerged as a non-invasive diagnostic tool for ATTR-CA. While its diagnostic accuracy is well-established for common variants, its effectiveness in detecting rare variants such as Glu54Gln remains unclear. Myocardial bisphosphonate uptake is suboptimal for certain TTR mutations, such as Ser97Tyr and Phe64Leu [4,5,7]. Therefore, our goal was to evaluate the diagnostic accuracy of scintigraphy for the Glu54Gln mutation. Although this mutation is less prevalent than others in endemic regions, it has been found to have a founding effect within the Romanian population. It is important to establish the accuracy of current diagnostic pathways not only for patients in Romania, but also for those of Romanian descent living in other countries. This study aimed to evaluate the diagnostic accuracy of bisphosphonate scintigraphy in Glu54GlnATTR-CA and evaluate its correlation with clinical and imaging findings.

## 2. Methods

### 2.1. Study Design and Population

A multicenter retrospective descriptive study was conducted, including 22 symptomatic patients with ATTR amyloidosis and 4 asymptomatic carriers who had the confirmed Glu54Gln variant. After a comprehensive evaluation that included electrocardiography, echocardiography, bisphosphonate scintigraphy, and clinical biomarkers, asymptomatic carriers were subsequently excluded from the study.

We included all known patients with the Glu54Gln ATTR mutation who performed bisphosphonate scintigraphy (BS) between February 2017 and January 2023 in a multicentric study involving four centers in Romania and one center in Florence, Italy. Patient identification was conducted through a referral network at the only Amyloidosis Referral Centre in Romania. All data were collected in a local database, which was later utilized to define the Glu54Gln cohort. It is important to note that active family screening was carried out to identify all relatives who were willing to undergo genetic testing. All patients in the study population were of Romanian descent, including the one patient diagnosed in Italy. The ethics committee approval for this study was obtained within the “Prof. Dr. C.C.Iliescu” Emergency Institute for Cardiovascular Diseases (code: 19962; date: 27 June 2023).

All patients signed an informed consent form regarding their agreement to process personal data for clinical and research purposes and agreed to the administration of the bisphosphonate radiotracer for diagnostic purposes, based on the local Ethical Committee regulations. There were no contraindications or delays in the injection of the radiopharmaceutical, and no patient was pregnant or breastfeeding, which represented exclusion criteria.

### 2.2. Data Collection

All patients underwent a comprehensive clinical assessment, including a detailed evaluation of both neurological and cardiological symptoms. Diagnostic procedures included serum biomarker analysis, a 12-lead electrocardiogram (ECG), and transthoracic echocardiography (TTE).

### 2.3. Serum Biomarkers

A complete blood count and basic biochemical screening were performed for all patients. Light chain (AL) amyloidosis was ruled out through the immunoelectrophoresis of serum and urinary proteins, as well as the quantification of serum-free light chains. Cardiac biomarkers, including N-terminal prohormone of brain natriuretic peptide (NT-proBNP) and high-sensitivity troponin I (TnI), were measured at the time of diagnosis.

### 2.4. Electrocardiography (ECG)

Baseline 12-lead surface ECG recordings were analyzed to assess QRS voltage and duration, as well as the presence of pseudo-infarction patterns. Additionally, atrial fibrillation and conduction abnormalities were documented.

### 2.5. Echocardiography

Standard transthoracic echocardiography (TTE) was performed in accordance with the European Association for Cardiovascular Imaging guidelines, using a Vivid scanner (GE Vingmed Ultrasound, Horten, Norway). Left ventricular (LV) systolic function was evaluated by calculating the LV ejection fraction (LVEF) using Simpson’s method in both 4- and 2-chamber views. Myocardial deformation was assessed through 2D speckle tracking and global longitudinal strain (GLS) calculation. Diastolic function was analyzed by assessing LV diastolic filling patterns and the ratio of E-wave to mitral annular diastolic velocity, and left atrial diameter was measured.

### 2.6. Bisphosphonates Scintigraphy (BS)

#### 2.6.1. Patient Preparation and Data Acquisition

Most patients (*n* = 25, 96%) underwent bisphosphonate scintigraphy using an intravenous administration of 99mTc-HDP (Oxidronate, Technescan™ HDP, Curium, Petten, The Netherlands) at an average dose of 700 MBq (±10%).One patient from Florence, Italy, underwent scintigraphy with 99mTc-HMDP (Oxidronate, OSTEOCIS^®^ Curium, Petten, The Netherlands). No dietary or medication restrictions were required for the procedure based on the patient’s existing comorbidities.

Following radiotracer administration, patients were instructed to hydrate adequately (1500–2000 mL of water) over 90 min and empty their bladder as needed, with mandatory voiding before the scan to minimize residual blood pool and soft tissue activity. Imaging began 120–150 min post-injection, using a gamma camera (Siemens e.cam, Dual Head Signature Series, 2007, Chicago, IL, USA) for the majority of patients *(n* = 23, 88%), and included whole body (WB) acquisition in anterior and posterior view, static chest acquisitions in anterior and posterior or lateral view, and single-photon emission computerized tomography (SPECT) of the chest. A minimum of 1000Kcounts/view was acquired for the WB imaging and 750Kcounts/view for the static planar images. The SPECT data included a wide field of view (FOV) with two active detectors in a 90° configuration. Acquisition with thirty-two frames/detector, rotation 180°, duration per frame was 25 s, and the starting angle was 45°. The chosen matrix was 64 × 64, and the zoom of 1.45 facilitated the analysis of the heart with the highest detection sensitivity. All patients were examined using a low-energy, high-resolution (LEHR) collimator, which featured a radionuclide energy peak of 140 keV and a 20% energy window. 

#### 2.6.2. Processing and Interpretation

Image processing involved reconstruction and evaluation across three planes: short axis, vertical long axis, and horizontal long axis. The scintigraphy interpretation followed a 3-step approach in line with established guidelines [6,8,9]: visual assessment for confirming ATTR-CA, semiquantitative grading based on myocardial tracer uptake, and the calculation of the heart-to-contralateral-uptake (H/CL) ratio. Additionally, a novel semiquantitative score, the heart-to-liver-uptake (H/L) ratio, was introduced, which, to our knowledge, has not previously been described in the literature. If myocardial uptake was confirmed on SPECT, the next step was semiquantitative visual grading (G), per Perugini’s classification [10]. If no myocardial uptake was observed on SPECT, the uptake was graded as G0. Myocardial tracer uptake was assessed relative to rib uptake using the following grading scale: G0—no myocardial uptake with normal bone uptake, in the absence of potential comorbidities at the level of the osteoarticular system; G1—myocardial uptake less than rib uptake; G2—myocardial uptake equal to rib uptake; G3—myocardial uptake greater than ribs with mild or moderately reduced uptake of the ribs. The diagnosis of ATTR-CA was considered for G2 and G3 uptake patterns.

For planar imaging, a circular region of interest (ROI) was placed over the heart (H), positioned left of the sternum, avoiding overlapping with costal structures. A mirrored contralateral (CL) ROI was placed in the right parasternal region to measure background activity. The H/CL ratio was calculated as the mean counts in the heart ROI, divided by the mean counts in the contralateral ROI.

Considering the variability of the uptake in the CL, we propose a new semiquantitative score, the H/L ratio. To assess background uptake, a new ROI of the same size as the heart ROI was mirrored in the liver region. The H/L ratio was analyzed in correlation with the H/CL ratio, providing a complementary semiquantitative measure of tracer distribution.

### 2.7. Histologic Testing

All patients had amyloid confirmation based on biopsy, most often performed from abdominal fat or salivary glands, with a histopathological examination in Congo Red staining in electron microscopy, with polarized light, to demonstrate the specific birefringence of amyloid fibrillar proteins.

### 2.8. Genetic Testing

All twenty-six patients underwent TTR gene sequencing, which confirmed the c.Glu54Gln (p.Glu74Gln per new nomenclature) variant.

### 2.9. Statistical Analysis

All statistical analyses were conducted using the SPSS software package (version 29, IBM Corp., Armonk, NY, USA). Continuous variables were expressed as the median [interquartile range] to account for skewness, while categorical variables were reported as a frequency (percentage). Comparisons between groups were performed using Student’s *t*-test or the Mann–Whitney U test for continuous variables, as appropriate. The Pearson chi-square test was used for categorical variables. A *p*-value < 0.05 was considered statistically significant. Where necessary, Bonferroni correction was applied for multiple comparisons. Graphs were generated using GraphPad Prism v.10 and IBM SPSS v.29.

## 3. Results

The study population comprised 22 Caucasian patients of Romanian descent, all of whom had the Glu54Gln TTR gene mutation. The mean age at diagnosis was 45 years, with the youngest patient being 30 years old and the oldest being 59 years old. Among the 22 symptomatic patients, one displayed an early cardiac phenotype, while the remaining 21 (95%) exhibited a mixed phenotype, which included both cardiac and neurological features. The main characteristics of the twenty-two symptomatic patients with ATTRGlu54Gln amyloidosis are presented in Table 1.

A comprehensive summary of twenty-six patients (both symptomatic and carriers) with ATTRGlu54Gln amyloidosis can be found in Supplementary Table S1.

### 3.1. Scintigraphy Findings

All twenty-two symptomatic patients, confirmed with the Glu54Gln mutation of TTR, had a positive BS for ATTR-CA: 4 (18%) had a Perugini score G2, while 18 pts (82%) had a G3 Perugini score.

All four healthy carriers with Glu54Gln TTR mutation had a G0 Perugini score, normal multimodality cardiac imaging, and electromyography.

### 3.2. Diagnostic Sensitivity

Using the high-grade uptake of 99mTc-HDP or HMDP on scintigraphy (G ≥ 2) as a diagnostic tool for ATTRGlu54Gln-CA, the method’s sensitivity was found to be 100% for both genotype-positive and phenotype-positive patients.

### 3.3. Semiquantitative Cardiac BS Assessment

**Figure 1:**
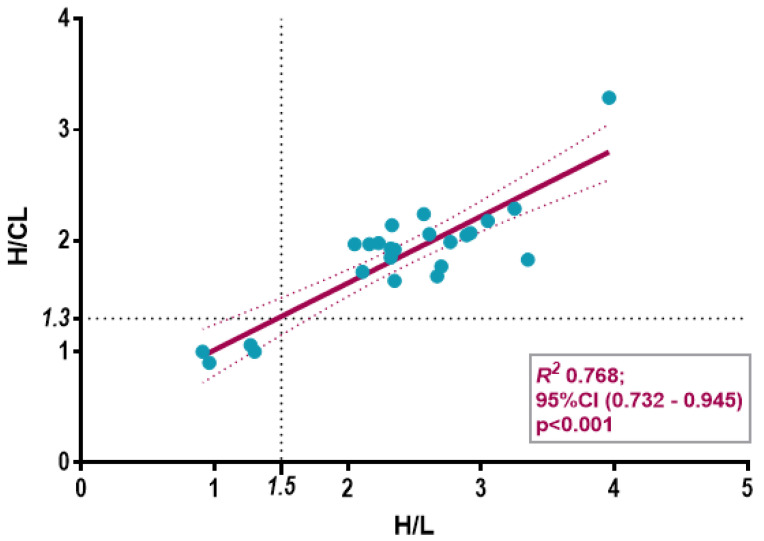
Scatter plot with linear regression analyses examining the relationships between H/CL and the H/L ratios, showing a strong positive correlation between the 2 parameters. The cut-off points of 1.5 for H/L and 1.3 for H/CL ratios are determined by the interpolation points derived from the linear regression curve.3.4. H/L vs. H/CL Ratios: and Correlation with Disease Severity.

We calculated radiotracer uptake in the heart using the H/CL ratio for all symptomatic patients (n = 21) and asymptomatic carriers (n = 4) for whom planar anterior chest images were available.When analyzing the relationship between H/CL and H/L ratios, our findings indicated a strong positive correlation (R^2^ = 0.768, *p* < 0.001). Based on interpolation points with the linear regression curve, we proposed a 1.5 cut-off point for this new parameter (Figure 1).

**Figure 2:**
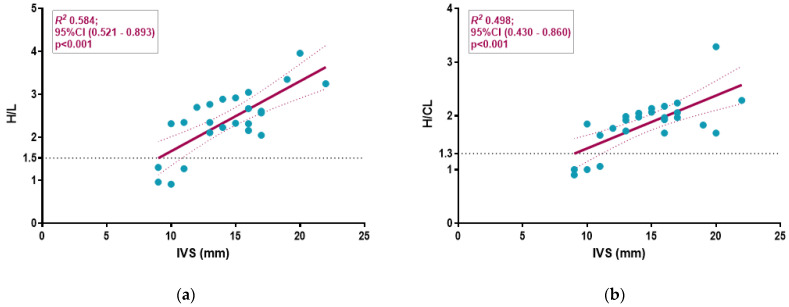
Scatter plot with linear regression analyses examining the relationships between the H/L ratio (**a**) and the H/CL ratio (**b**) and the thickness of the interventricular septum (IVS). The H/L ratio (**a**) showed a strong positive correlation with the increase in IVS thickness, like the H/CL ratio (**b**).

**Figure 3:**
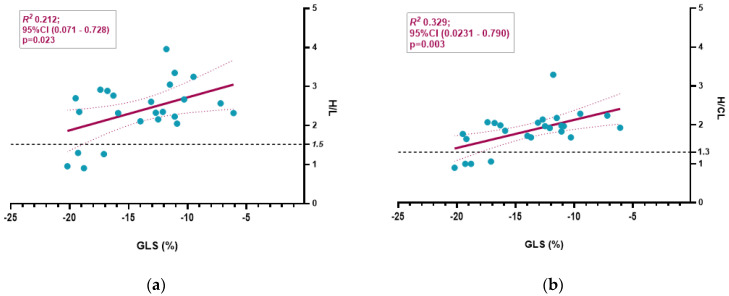
Scatter plot with linear regression analyses examining the relationships between the H/L ratio (**a**) and the H/CL ratio (**b**) and global longitudinal strain (GLS). The H/L ratio (**a**) showed a weak correlation with GLS alteration, whereas the H/CL ratio had a better performance (**b**).

We analyzed the relationship between echocardiographic parameters and both H/CL and H/L ratios (Table 2; Figure 2 and Figure 3).

Supplementary Table S2 present the differences in the Perugini degree of uptake (G), H/CL and H/L ratios, along with the cardiac patterns observed in SPECT and the extracardiac WB uptake.

The H/CL ratio values varied from 1 to 3.29, while the H/L ratio values ranged from 0.91 to 3.96 among the same patients.

### 3.4. Extracardiac WB Uptake and SPECT

Of the twenty-two patients with the Glu54Gln mutation and CA confirmed by scintigraphy, none had extracardiac uptake of the radiotracer.

The qualitative and semiquantitative evaluation of the planar studies identifies radiotracer accumulation in the left ventricle (LV) walls, as confirmed by SPECT acquisition. A diffuse, low-intensity accumulation was observed at the right ventricle (RV) wall level in 100% of patients with G2/G3 uptake degrees (see Supplementary Table S2).

Compared to healthy carriers, patients with Perugini Grade 3 uptake had more severe cardiac involvement, characterized by higher NT-proBNP levels, compared to Grade 2, more severe biventricular hypertrophy with increased interventricular septum (IVS) thickness, with more altered LV function, with reduced GLS as seen in Table 2.

Low QRS voltage was observed only in the G3 BS uptake group. Additionally, the G3 cardiac uptake group exhibited a higher prevalence of right bundle branch block (RBBB) and pseudo-infarction patterns (PIP). When analyzing the relationship of echocardiographic parameters with the two ratios, both H/L and H/CL demonstrated a similar strong positive correlation with IVS thickening (R^2^ = 0.584, *p* < 0.001 and R^2^ = 0.498, *p* < 0.001) (Figure 2).

Neither the H/L nor H/CL ratio had a significant correlation with NT-proBNP (R^2^ = 0.127, *p* = 0.10 for H/L; R^2^ = 0.094, *p* = 0.154 for H/CL, respectively) and Troponin I (R^2^ = 0.11, *p* = 0.641 for H/L; R^2^ = 0.003, *p* = 0.798 for H/CL, respectively).

The correlation of semiquantitative BS parameters with GLS was weak for the H/L ratio (R^2^ = 0.212, *p* = 0.023), while the H/CL ratio performed better (R^2^ = 0.329, *p* = 0.003) (Figure 3).

### 3.5. Case Highlights

**Figure 4:**
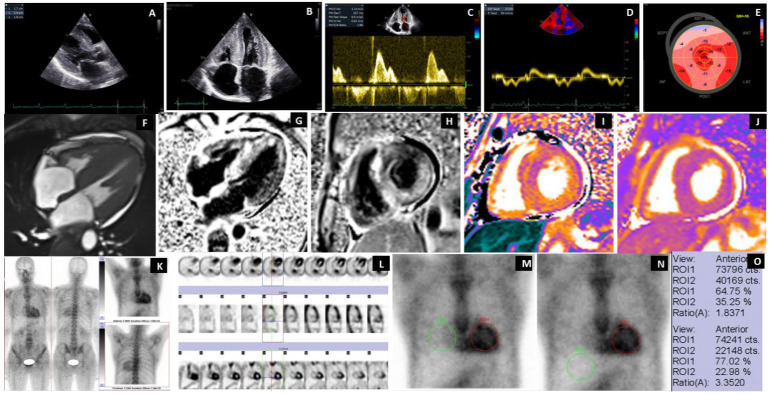
Representative case of G3 uptake in an ATTRGlu54Gln CA patient with mixed phenotype. Transthoracic echocardiography (**A**–**E**). Parasternal long axis diastolic frame (**A**) and apical 4 chambers systolic frame (**B**) of a very typical, advanced patient showing severe concentric LV hypertrophy, RV free wall hypertrophy, dilated atria, thickened atrioventricular valves, and a trace of pericardial effusion. Pulsed wave Doppler with the sample volume located at the tip of the mitral leaflets (**C**) and Tissue Doppler echocardiography with the sample volume located at the base of the septal mitral annulus (**D**), revealing advanced grade II diastolic dysfunction with an E wave which is taller than the A wave and severe longitudinal impairment of both systolic and diastolic myocardial velocities and an E/e’ of 23. Automated function imaging derived from the speckle tracking echocardiography (**E**) revealed a severely impaired GLS with the apical sparing pattern of the Bull’s eye view. Contrast-enhanced cardiac magnetic resonance (**F**–**J**). Cine (balanced SSFP) 4-chamber sequences (**F**) revealed the same findings as the transthoracic echocardiography. Late gadolinium enhancement sequences in 4 chambers and basal short axis show diffuse, transmural LGE, which also involves the papillary muscles, the RV-free wall, and the atrial walls with abnormal gadolinium kinetics (**G**,**H**). Noncontrast T1 mapping acquired on a 1.5 T scanner revealed a severely elevated native T1 of 1265 ms, corresponding to a severe extracellular expansion (ECV 63%) (**I**). A slightly increased native T2 (56 ms) reflecting a degree of myocardial edema (**J**). Bisphosphonate scintigraphy (**K**–**O**). Planar Whole-Body acquisition in the anterior and posterior view from the vertex to the proximal 1/3 of the thighs. Static acquisition at the chest in the anterior and posterior views. Diffusely increased uptake was observed at the heart, with intensity greater than the costal one, with a Perugini visual uptake score of G3 (**K**). Acquisition of SPECT at the chest, with the delimitation of the LV walls and free wall of the RV, after the reconstruction and display of the transverse, sagittal, and coronal slices (**L**). Selection of ROIs from the LV level and the contralateral region where the RV overlaps (**M**). Selection of ROIs at the level of the LV and the right subdiaphragmatic, in the liver region without including the RV (**N**). The difference in the semiquantitative score of the H/CL (*n* = 1.83) and H/L (*n* = 3.35) ratios is due to the overlap of the RV. (**O**) ECV = extracellular volume; GLS = global longitudinal strain; LGE = late gadolinium enhancement; LV = left ventricle; ROI = region of interest; RV = right ventricle; SPECT = single-photon emission computer tomography; SSFP=steady-state free precession.

Figure 4 shows a representative patient with ATTRGlu54Gln CA with mixed phenotype cardiac, neurological, and autonomic system involvement, with ECG and echocardiographic findings suggesting cardiac amyloidosis, with diffuse transmural late gadolinium enhancement (LGE) pattern on cardiac magnetic resonance imaging (cMRI) and with a myocardial uptake of 99mTc-HDP on BS, with the maximal semiquantitative score for ATTR-CA (G3 score). The anterior static views of the chest show the ROI from the level of the heart and in the mirror, respecting the same sizes at the level of the contralateral and the liver regions. The average number of counts and the ROI ratios were calculated.

**Figure 5:**
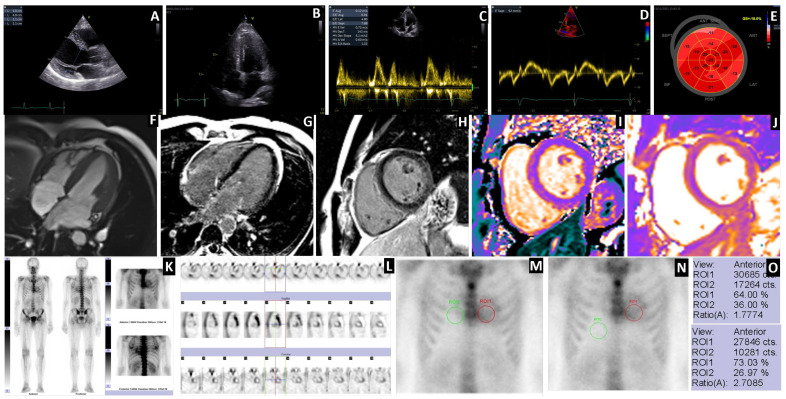
Youngest patient with early cardiac involvement with G2 score. Transthoracic echocardiography (**A**–**E**). Parasternal long axis diastolic frame (**A**) and apical 4-chamber systolic frame (**B**) of the youngest patient presented with normal cavity sizes and function. Pulsed wave Doppler with the sample volume located at the tip of the mitral leaflets (**C**) and Tissue Doppler echocardiography with the sample volume located at the base of the septal mitral annulus (**D**), revealing normal diastolic and longitudinal function. Automated function imaging derived from the speckle tracking echocardiography (**E**), revealing a normal GLS with a normal pattern of the Bull’s eye view. Contrast-enhanced cardiac magnetic resonance (**F**–**J**). Cine (balanced SSFP) 4-chamber sequences (**F**) revealed the same findings as the transthoracic echocardiography. Late gadolinium enhancement sequences in 4 chambers and basal short axis show only minor, subendocardial LGE, more prominent on the right side of the interventricular septum with normal gadolinium kinetics (**G**,**H**). Noncontrast T1 mapping acquired on a 1.5 T scanner revealed a less elevated native T1 of 1126 ms corresponding to a less severe extracellular expansion (ECV 38%) (**I**). A normal native T2 (44 ms), demonstrating no evidence of myocardial edema (**J**). Bisphosphonate scintigraphy (**K**–**N**). Planar Whole-Body acquisition in the anterior and posterior incidence from the vertex to the soles. Static acquisition at the chest level in the anterior and posterior views showing diffusely increased uptake at the heart level, of the same intensity as the costal one, with a Perugini visual uptake score of G2 (**K**). Acquisition of SPECT at the chest level, with the delimitation of the LV walls and free wall of the RV, after the reconstruction and display of the transverse, sagittal, and coronal slices (**L**). Selection of ROIs from the LV level and the contralateral region where the RV overlaps (**M**). Selection of ROIs at the LV level and liver regions, without including the RV (**N**). The difference in the semiquantitative score of the H/CL (*n* = 1.77) and H/L (*n* = 2.70) ratios is due to the overlap of the RV. (**O**) ECV = extracellular volume; GLS = global longitudinal strain; LGE = late gadolinium enhancement; LV = left ventricle; ROI = region of interest; RV = right ventricle; SPECT = single-photon emission computer tomography; SSFP=steady-state free precession.

The oligosymptomatic patient (30 y, M) with a G2 score at BS had an IVS of 12 mm and an LGE with a subendocardial pattern. Early cardiac involvement was accompanied by carpal tunnel syndrome (CTS) but without polyneuropathy or autonomic nervous system involvement. This was the youngest patient with an early cardiac phenotype with the ATTRGlu54Gln mutation present, demonstrating the diagnostic value of BS (Figure 5).

## 4. Discussion

### 4.1. Prevalence of ATTRGlu54Gln Amyloidosis in Romania

In Romania, the most frequent TTR mutation is Glu54Gln, described in 2012 by Coriu et al. [3], with a common geographic origin in northeastern Romania in the village of Todiresti, Suceava County [11]. Jercan et al. reported the first 18 patients with this genotype, presenting a mixed, cardiological and neurological phenotype with an onset in the 4th decade of life. The known prevalence of ATTRv amyloidosis in Romania is 1.02 per million (0.76 per million for the ATTRGlu54Gln mutation), and in Suceava county, it is 2.39 per 100,000 inhabitants for the same TTR mutation [11].

### 4.2. Diagnosis of ATTR Amyloidosis

Endomyocardial biopsy and mass spectrometry remain the gold standard for diagnosing and typing amyloid fibrillar protein [9,12]. However, these methods are invasive and often inaccessible, limiting their widespread use. As an alternative, extracardiac biopsy—from salivary glands, abdominal fat, digestive mucosa, or kidney tissue—combined with immunohistochemistry or immunoelectron microscopy is commonly employed in specialized centers. For a non-biopsy diagnosis, three key diagnostic criteria are used: 1. cardiac imaging findings (echocardiography and MRI); 2. bisphosphonate scintigraphy (BS) with a Perugini visual grade G2 or G3; and 3. exclusion of monoclonal proteins via serum and urine electrophoresis with immunofixation (SPIE, UPIE) and free light chain (FLC) analysis [9,12,13].

### 4.3. Binding of Bisphosphonates to TTR Amyloid Fibrillar Proteins

The exact mechanism of bisphosphonate binding in cardiac imaging remains unclear but is believed to be linked to calcium content within amyloid deposits [1,14].

Another hypothesis suggests that the binding affinity depends on the type of amyloid fibrils. Type A fibrils consist of a mixture of C-terminal and full-length ATTR fibrils, while type B fibrils contain only full-length ATTR fibrils. These fibril subtypes correlate with different clinical phenotypes. Studies indicate that patients with Type A fibrillar deposits demonstrate high bisphosphonate uptake (99mTc-DPD scintigraphy), while those with Type B fibrils do not accumulate bisphosphonates at the myocardial level [15,16].

### 4.4. Diagnostic Accuracy in Detecting Glu54Gln ATTR-CA

Our study showed an excellent diagnostic accuracy of BS for detecting Glu54Gln ATTR-CA. A Perugini visual score of G2/G3 was 100% sensitive for identifying myocardial involvement, and negative scans effectively ruled out cardiac amyloidosis in asymptomatic carriers. While some TTR mutations (e.g., Ser97Tyr, Phe64Leu) have been associated with lower scintigraphy sensitivity, BS demonstrated 100% diagnostic accuracy in ATTRGlu54Gln CA patients, confirming its utility even in early-stage or oligosymptomatic case [7,13].

Moreover, the Perugini score strongly correlated with disease severity. Patients with G3 scores exhibited more severe cardiac hypertrophy, increased biomarkers, and reduced myocardial strain, reflecting advanced disease stages. The diagnostic sensitivity of BS for the Glu54Gln mutation was comparable to other common TTR mutations [13]. Combining Perugini G2/G3 scoring with monoclonal protein exclusion resulted in a 100% specificity and positive predictive value for ATTR-CA.

### 4.5. Conceptual Differences Between H/CL and H/L Ratios

Semiquantitative interpretation of BS in the late acquisition phase is affected by various factors, including the time interval between radiotracer administration and imaging, as well as the criteria used to define the ROI for uptake measurement. The traditional H/CL ratio may be affected by factors such as right ventricular overlap, inclusion of costal arches, and increased background activity in the right lung due to reduced estimated glomerular filtration rate (eGFR) or insufficient waiting time. Given these limitations, we propose the H/L ratio as a potentially more reliable metric for evaluating myocardial bisphosphonate uptake in cardiac amyloidosis.

The H/CL ratio represents the myocardial retention (H) of the radiotracer, from which the contralateral region (CL), located parasternally on the right side and representing background tissue absorption, is subtracted. Established thresholds indicate that an H/CL ratio ≥ 1.5 at 1 h can accurately identify ATTR cardiac amyloidosis when myocardial uptake is confirmed on SPECT and systemic AL amyloidosis is excluded [6,8]. Additionally, the 2021 Addendum by Dorbala et al. suggested that an H/CL ratio ≥ 1.3 at 3 h could be diagnostic, as the radioactive background decreases while the image contrast improves [9]. However, the H/CL ratio lacks diagnostic value when there is no myocardial uptake on SPECT (Perugini Grade 0) or in cases of Grade 2 or 3 uptake, where the H/CL ratio generally aligns with visual assessment. In cases of discordance or equivocal semiquantitative grades (e.g., G1 or G2), it can help with interpretation [9,13].

However, a major limitation arises from overestimated right parasternal uptake due to RV activity, particularly in patients with ventricular hypertrophy secondary to amyloidosis, which can lead to underestimation of myocardial involvement. The H/L ratio uses the liver (L) as the reference region, offering a more stable background for radiotracer activity. Since cardiac ATTR amyloidosis affects all heart structures, though predominantly accumulating in the LV due to its thicker walls, RV uptake should not be overlooked. However, using the liver as a reference instead of the contralateral ROI minimizes interference from RV activity and other anatomical structures.

The involvement of the RV can predict the most severe course of the disease and is useful to achieve an early diagnosis [17]. A recent study indicated that diffuse, rather than focal RV uptake of bisphosphonate tracers, serves as an independent prognostic marker at diagnosis [18]. At the same time, it underlines the diffuse nature of amyloid infiltration in the cardiac walls. A SPECT analysis showed diffuse RV uptake in all 22 patients studied, indicating a severe progression of transthyretin amyloidosis, particularly in those with the Glu54Gln mutation. However, we did not perform any analyses regarding the regional characteristics of RV uptake.

While amyloid involvement in the atria and valvular structures cannot be ruled out, detection remains challenging with the current SPECT resolution. Future advancements, such as 11C-labeled Pittsburgh compound B (PiB) and thioflavin analog tracers (e.g., 18F-florbetapir, 18F-florbetaben, 18F-flutemetamol), combined with digital PET-CT, may enhance detection beyond the ventricles [19,20]. Although unlike the radiotracers used in SPECT, amyloid PET-CT radiotracers do not differentiate ATTR from AL amyloidosis, a new pan-amyloid radiotracer, 124I-evuzatamide, demonstrates similar uptake in AL-CA and higher uptake in ATTR compared to 18F-florbetapir. [21].

Some advantages of the H/L ratio may include reduced variability in background activity, minimization of artifacts, and improved sensitivity for diffuse uptake. The hepatic region is less likely to include confounding anatomical structures (e.g., RV, ribs, sternum) or overlapping blood pool radioactivity, leading to more consistent background measurements. Moreover, the liver is less prone to contamination from residual radiotracer in the blood pool or skeletal uptake, reducing the risk of falsely elevated background counts. In cases of extensive myocardial involvement, the H/CL ratio may underestimate uptake due to spillover effects, whereas the H/L ratio could provide better contrast and reflect true myocardial retention.

Our study brought preliminary findings suggesting that the H/L ratio could correlate more strongly with Perugini grades and interventricular septum thickness than the H/CL ratio. In our analysis, none of the parameters showed a correlation with cardiac biomarkers, while the relation to global longitudinal strain (GLS) was suboptimal. These results require larger datasets for confirmation.

Galat et al. proposed an alternative heart-to-mediastinum (H/M) ratio with early scanning at 10 min [22]. Given that the liver background is lower than the contralateral region, early image acquisition may be sufficient for reliable H/L ratio assessment, potentially reducing required waiting times.

### 4.6. Hepatic Uptake of Bisphosphonates

The diffuse hepatic uptake of bisphosphonates can occur in hepatic necrosis caused by hepatotoxic medications such as amphotericin B, methotrexate, epirubicin, and certain antibiotics. This uptake may also arise due to respiratory failure from bilateral miliary pulmonary metastases [23]. Other potential causes include aluminum intoxication in chronic dialysis patients and altered metabolism of phosphate and calcium. [23,24].

Bisphosphonate focal uptake is typically linked to liver metastases or hepatic calcifications in patients with hyperparathyroidism or following liver transplantation [24,25]. Uptake in the hepatosplenic reticuloendothelial system may result from improper radiopharmaceutical preparation or contamination with colloidal impurities [26].

In a study by Malka et al. involving 247 patients with cardiac amyloidosis, none of the patients with transthyretin amyloid cardiomyopathy exhibited hepatic uptake, and three patients with light chain amyloidosis showed hepatic bisphosphonate uptake [27].

In our cohort, all patients had normal levels of transaminases and total bilirubin, and their tumor and viral markers were also within normal limits. None of the patients demonstrated focal or diffuse hepatic uptake of bisphosphonates. Moreover, previous studies that assessed hepatic uptake utilized 99mTc-MDP (technetium-methylene diphosphonate) for nuclear imaging [23,24], a radiopharmaceutical not recommended for diagnosing transthyretin amyloidosis. The hepatic uptake of bisphosphonate is rare. However, when it does occur, the use of the H/L ratio is not recommended.

### 4.7. Study Limitations

Our study focused on a small population, so the accuracy of the statistical tests may be limited to interpreting trends rather than reporting statistical significance. Furthermore, there are certain limitations of the H/L ratio, such as its susceptibility to abdominal factors. Liver or bowel tracer pooling, particularly in patients with hepatic dysfunction, could introduce variability.

Although promising, the H/L ratio requires further validation to establish reproducibility, diagnostic thresholds, and clinical relevance. Unlike the extensively studied H/CL ratio, the H/L ratio lacks established benchmarks for different amyloid subtypes and mutations, necessitating further comparative quantitative studies.

## 5. Conclusions

BS shows 100% sensitivity in patients with symptomatic ATTRGlu54Gln CA, as well as a high value in identifying mutation carriers, making it a reliable tool for carrier follow-up. Diffuse cardiac uptake from both ventricles, associated with this genotype, suggests a severe cardiac phenotype, with a reserved prognosis. Our study proposed a new semiquantitative parameter for BS interpretation, the H/L ratio, potentially addressing key limitations of the traditional H/CL ratio. By using the liver as a reference, the H/L ratio minimizes variability, reduces artifacts, and may improve the sensitivity for detecting myocardial involvement. However, additional research is needed to confirm its diagnostic accuracy, standardize reference values, and evaluate its clinical utility in broader patient populations.

## Figures and Tables

**Table 1:** Summary of symptomatic patients’ demographics and clinical features.

Clinical Characteristics	ATTRGlu54Gln Symptomatic Patients’ Data (*n* = 22)
Age at diagnosis (y); median [IQR]	45 [43–48]
Female, *n* (%)	15 (68)
Family history of ATTR amyloidosis, *n* (%)	21 (95.4)
BMI (kg/m^2^) [IQR]	23.4 [19.4–25.9]
Proband status *n* (%)	22 (100%)
Birthplace: Suceava county, Romania *n* (%)	21 (95.45)
NT-proBNP (pg/mL) [IQR]	1457 [94.8–3707.5]
TnI (ng/mL) [IQR]	0.017 [0.01–0.02]
Cardiac involvement *n* (%)	22 (100%)
Phenotype n (%)	
Cardiac incipient	1 (5%)
Mixed phenotype (cardiac + neurologic)	21 (95%)
NYHA functional class n (%)	
No HF symptoms reported	6 (27.4%)
I	4 (18.1%)
II	9 (40.9%)
III	3 (13.6%)
IV	0
Syncope, *n* (%)	3 (13.6%)
ICD, *n* (%)	2 (9%)
Pacemaker, *n* (%)	2 (9%)
Autonomic involvement, *n* (%)	20 (91%)
Peripheral neuropathy, *n* (%)	21 (95%)
Bilateral carpal tunnel syndrome, *n* (%)	19 (86%)

Legend: values are *n* (%), or median [IQR]= interquartile range; I–IV=NYHA functional classes of heart failure; ATTR = transthyretin amyloid; BMI = body mass index; HF = heart failure; NYHA = New York Heart Association; NT-proBNP = N-terminal (NT)-pro hormone B-type natriuretic peptides; TnI = troponin I; ICD = implantable cardioverter-defibrillator.

**Table 2:** Perugini visual uptake score and its relationship with patient phenotype.

	G0 Score at BS (Carriers 4pts.)	G2 Score at BS (4 pts.)	G3 Score at BS (18 pts.)	*p*-Value
Age at Diagnosis (y)	33.5 [30–34]	42.5 [35–45]	45 [43–48] ^#^	<0.001
Male	3 (75)	1 (25)	6 (33.3)	NS
NYHA II/III	0	1 (25)	11 (61.1)	0.05
ICD	0	0	2 (11.1)	NS
Pacemaker	0	0	2 (11.1)	NS
NT-proBNP (pg/mL)	17.25 [12.2–22.15]	512.0 [76.5–2229]	1632.5 [884–4218.5]	NS
TnI (ng/mL)	0.005 [0.001–0.02]	0.01 [0.0045–0.0165]	0.02 [0.01–0.0255]	NS
Normal ECG	4 (100)	2 (50)	3 (16.6)	NS
Low QRS Voltage	-	0	11 (61.1)	0.002
Voltage/mass ratio	0.33 [0.24–0.39]	0.13 [0.06–0.22]	0.10 [0.06–0.185]	NS
Pseudo-infarction pattern	-	1 (25)	11 (61.1)	0.05
RBBB	-	0	6 (33.3)	NS
AVB				NS
1st AVB	-	2 (28.5)	4 (22.2)	
2nd AVB	-	2	1 (5.5)	
3rd AVB	-	0	2 (11.1)	
AF	-	0	2 (11.1)	NS
NSVT	-	1 (25)	5 (27.7)	NS
IVS (mm)	9.5 [9–10.5]	13.5 [11.5–17.5]	16 [13.5–17] ^#^	0.003
LVPW (mm)	9.5 [9–11]	12.5 [11–15]	14 [12–16] ^#^	0.02
LVED Diameter (mm)	45.5 [44–47]	44.5 [40.5–50]	42 [38–48.5]	NS
LVMi (g/m^2^)	73.0 [63.25–81.32]	139.7 [116.3–173.0]	146.3 [116.7–166.1] ^#^	0.01
LVED Volume (mL)	96 [79–118]	74.5 [62–91]	77 [67.5–94.5]	NS
LVEF (%)	58.5 [58–60]	62 [59.5–65]	55 [46.5–58.5]	0.049
LV GLS	−19.0 [−19.7–−17.9]	−15.9 [−19.5–−11.5]	−12.5 [−15.2–−10.6] ^#^	0.002
LA Diameter (mm)	33.5 [32.5–36.5]	35 [34–39]	41 [38–43]	NS
RV FW (mm)	4.0 [4–4.5]	5.5 [4.5–6]	7 [6–8] ^#^	0.004
High LV filling pressure	-	2 (50)	11 (61)	NS
Pericardial fluid (Y/*n*)	-	0	4 (22.2)	NS
H/CL	1 [0.95–1.03]	1.77 [1.72–1.81] ^#^	1.99 [1.92–2.14] ^#^	<0.001
H/L	1.12 [0.94–1.29]	2.42 [2.37–2.56] ^#^	2.59 [2.32–3.25] ^#^	<0.001
Autonomous involvement	-	3(75)	17 (94)	<0.001
SMP	-	3 (75)	18 (100)	0.05
Carpal tunnel syndrome	1 (25)	3 (75)	16 (89)	0.01

Values are median [interquartile range] or *n* (%). ^#^ *p* < 0.05 vs. group G0. (1st/2nd/3rd AVB) = 1st/2nd/3rd degree atrioventricular block; AF = atrial fibrillation; BS = bisphosphonates scintigraphy; ECG = electrocardiography; H/CL = heart-to-contralateral-uptake ratio; H/L = heart-to-liver-uptake ratio; LV = left ventricle; LVED = left ventricle end diastolic; LV GLS = left ventricle global longitudinal strain; ICD = implantable cardioverter-defibrillator; IVS = interventricular septum; LVEF = left ventricle ejection fraction; LVMi = left ventricle mass Index; LVPW = left ventricle posterior wall; LA = left atrial; NS = not significant; NSVT = non-sustained ventricular tachycardia; NYHA = New York Heart Association Functional Classification of Heart Failure; NT-proBNP = N Terminal-prohormone Brain Natriuretic Peptide; *p*-value = statistical significance; QRS= QRS complex in electrocardiography; RBBB = right bundle branch block; RV FW = right ventricle free wall; SMP = sensory-motor polyneuropathy; TnI = troponin I.

## Data Availability

The original contributions presented in this study are included in the article. Further inquiries can be directed to the corresponding author.

## Supplementary Materials

The following supporting information can be downloaded at: https://www.mdpi.com/article/10.3390/jcm14113734/s1, Table S1: Scintigraphy results and correlations with clinical parameters; Table S2: Qualitative and semi-quantitative scintigraphic parameters.

## Abbreviations

The following abbreviations are used in this manuscript:

99mTc	metastable technetium
AL	amyloid light chain
ATTR	amyloid transthyretin
ATTR-CA	amyloid transthyretin-cardiomyopathy
BS	bisphosphonate scintigraphy
cMRI	cardiac magnetic resonance imaging
DPD	3,3-diphosphono-1,2-propanodicarboxylic acid
ECG	electrocardiography
G	cardiac uptake Perugini score
Glu54Gln	glutamic acid 54 glutamine
GLS	global longitudinal strain
H/CL	heart to contralateral uptake
HDP	oxidronate
HMDP	oxidronate
H/L	heart to liver uptake
LV	left ventricle
LEHR	low energy and high resolution
LVEF	left ventricular ejection fraction
MDP	methylene diphosphonate
NT-proBNP	N-terminal prohormone of brain natriuretic peptide
PET	positron emission tomography
PIP	pseudo-infarction pattern
ROI	region of interest
RBBB	right bundle branch block
RV	right ventricle
SPECT	single-photon emission computed tomography
TnI	high-sensitivity troponin I
TTE	transthoracic echocardiography
TTR	transthyretin
WB	whole body
