# Supplementary material for: Diagnostic Accuracy of Bisphosphonate Scintigraphy in Glu54GlnATTR Cardiomyopathy
Source: J Clin Med. 2025 May 26;14(11):3734. doi: 10.3390/jcm14113734 (PMC12155782; doi:10.3390/jcm14113734)
Supplement: Supplementary file 1 [file jcm-14-03734-s001.zip › jcm-3622152-supplementary.pdf]

Supplementary materials

Table S1. Scintigraphy results and correlations with clinical parameters.

Pts.	Sex	AD (y)	Proband/ carrier	IVS (mm)	LVEF (%)	GLS (%)	LAD (mm)	BS Grade	ECG	Neurological involvement
1BA	F	50	P	15	58	-12,7	45	3	LQV, RBBB	AN, PN, CTS
2CP	M	51	P	17	31	-7,2	38	3	LQV, RBBB, LAH, AF, PIP	AN, PN
3BC	F	47	P	13	59	-16,3	41	3	Normal	AN, PN, CTS
4SA	M	49	P	14	61	-16,8	35	3	LQV	AN, PN, CTS
5RL	F	47	P	16	45	-10.3	41	3	LQV, LAH, AF, PIP	AN, PN, CTS
6SC	F	49	P	17	47	-10,9	46	3	LQV, RBBB, LAH, PIP, 1stAVB	AN, PN, CTS
7TV	M	42	P	20	55	-11,8	43	3	RBBB, PIP, 3rdAVB, NSVT	AN, PN, CTS
8SM	M	32	C	11	59	-17.1	39	0	Normal	Normal
9NG	F	41	P	16	33	-6.1	42	3	LQV, RBBB, LAH, PIP	AN, PN, CTS
10PA	F	34	C	9	61	-20.2	32	0	Normal	CTS
11IM	M	43	P	22	58	-9.5	43	3	LQV, LAH, PIP, 1stAVB, NSVT	AN, PN, CTS
12HE	F	40	P	10	59	-15.9	34	2	Normal	AN, PN, CTS
13MV	F	35	P	15	63	-17.4	31	3	Normal	AN, PN
14CM	F	59	P	16	62	-12.5	42	3	LQV, RBBB, PIP, 3rdAVB	AN, PN, CTS
15TA	M	34	C	10	58	-18.8	33	0	Normal	Normal
16IC	M	44	P	13	60	-12.1	41	3	LQV	AN, PN, CTS
17SL	F	43	P	13	55	-14	41	3	LQV, PIP	AN, PN, CTS
18IL	F	44	P	11	55	-19.2	35	3	Normal	PN, CTS
19GS	M	30	P	12	60	-19.5	38	2	Normal	Normal
20BC	F	55	P	19	51	-11.1	38	3	LQV, PIP, 1stAVB, NSVT	AN, PN, CTS
21HM	F	45	P	16	46	-11.5	65	3	1stAVB NSVT	AN, PN, CTS

22BN	F	47	P	20	64	-13.7	36	2	LAH, PIP, 1stAVB, NSVT	AN, PN, CTS
23PC	M	28	C	9	58	-19.3	34	0	Normal	Normal
24PG	M	48	P	17	50	-13.1	34	3	PIP, 2ndAVB NSVT	AN, PN, CTS
25CD	F	41	P	14	45	-11.1	42	3	PIP	AN, PN, CTS
26FI	F	45	P	15	67	-14.8	42	2	1stAVB	AN, PN, CTS

Legend: (1st/2nd/3rd AVB)=1st/2nd/3rd degree atrio-ventricular block; AD=age at diagnosis; AF=atrial fibrillation; AN=autonomic neuropathy; C=carrier; CTS=carpal tunnel syndrome; cBS grade=uptake Perugini grading in cardiac bisphosphonates scintigraphy; ECG=electrocardiography; GLS=global longitudinal strain; IVS=interventricular septum; LVEF=left ventricular ejection fraction; LAD=left atrial diameter; LAH=left anterior hemiblock; LQV=low QRS voltages; NI=neurological involvement; PN=sensory-motor polyneuropathy;Pts=patient; RBBB=right bundle branch block; NSVT=non-sustained ventricular tachycardia; PIP-pseudo-infarction pattern; P=proband;

Table S2. Qualitative and semi-quantitative scintigraphic parameters.

Pts #	Grade	H/CL	H/L	SPECT Uptake	Extracardiac WB Uptake
1/ B.A.	3	2.14	2.33	Diffuse LV+RV	0
2/ C.P.	3	2.24	2.57	Diffuse LV+RV	0
3/B.C.	3	1.99	2.77	Diffuse LV+RV	0
4/S.A.	3	2.05	2.89	Diffuse LV+RV	0
5/R.L	3	1.68	2.67	Diffuse LV+RV	0
6/S.C.	3	1.97	2.05	Diffuse LV+RV	0
7/T.V	3	3.29	3.96	Diffuse LV+RV	0
8/N.G.	3	1.93	2.32	Diffuse LV+RV	0
9/I.M.	3	2.29	3.25	Diffuse LV+RV	0
10/H.E.	2	1.85	2.32	Diffuse LV+RV	0
11/M.V.	3	2.07	2.92	Diffuse LV+RV	0
12/C.M.	3	1.97	2.16	Diffuse LV+RV	0
13/I.C.	3	1.92	2.35	Diffuse LV+RV	0
14/S.L	3	1.72	2.11	Diffuse LV+RV	0
15/I.L.	3	1.64	2.35	Diffuse LV+RV	0
16/G.S.	2	1.77	2.70	Diffuse LV+RV	0
17/B.C.	3	1.83	3.35	Diffuse LV+RV	0

18/H.M.	3	2.18	3.03	N.A.	0
19/B.N.	2	1.68	2.42	Diffuse LV+RV	0
20/P.G.	3	2.06	2.61	Diffuse LV+RV	0
21/C.D.	3	1.98	2.23	Diffuse LV+RV	0
22/F.I.	2	-	-	Diffuse LV+RV	0
23/S.M./Carrier	0	1.06	1.27	Absent	0
24/P.A./Carrier	0	0.90	0.96	Absent	0
25/T.A./Carrier	0	1.00	0.91	Absent	0
26/P.A./Carrier	0	1.00	1.30	Absent	0

Legend: Grade=qualitative Perugini uptake score; H/CL=heart to contralateral uptake ratio; H/L=heart to liver uptake ratio; SPECT=single photon emission computer tomography; LV=left ventricle; RV=right ventricle; WB=whole-body acquisition.
